# Pancreatic Morphology, Immunology, and the Pathogenesis of Acute Pancreatitis

**DOI:** 10.3390/biomedicines12112627

**Published:** 2024-11-17

**Authors:** Tudorel Mihoc, Silviu Constantin Latcu, Cosmin-Ciprian Secasan, Vlad Dema, Alin Adrian Cumpanas, Mircea Selaru, Catalin Alexandru Pirvu, Andrei Paul Valceanu, Flavia Zara, Cristina-Stefania Dumitru, Dorin Novacescu, Stelian Pantea

**Affiliations:** 1 Doctoral School, Victor Babes University of Medicine and Pharmacy Timisoara, E. Murgu Square, No. 2, 300041 Timisoara, Romania; mihoc.tudorel@umft.ro (T.M.); vlad.dema@umft.ro (V.D.); 2 Department X, General Surgery II, Discipline of Surgical Emergencies, Victor Babes University of Medicine and Pharmacy Timisoara, E. Murgu Square, No. 2, 300041 Timisoara, Romania; selaru.mircea@umft.ro (M.S.); pirvu.catalin@umft.ro (C.A.P.); valceanu.andrei@umft.ro (A.P.V.); pantea.stelian@umft.ro (S.P.); 3 Department XV, Discipline of Urology, Victor Babes University of Medicine and Pharmacy Timisoara, E. Murgu Square, No. 2, 300041 Timisoara, Romania; cosmin.secasan@umft.ro (C.-C.S.); cumpanas.alin@umft.ro (A.A.C.); 4 Department II of Microscopic Morphology, Victor Babes University of Medicine and Pharmacy Timisoara, E. Murgu Square, No. 2, 300041 Timisoara, Romania; flavia.zara@umft.ro (F.Z.); cristina-stefania.dumitru@umft.ro (C.-S.D.); novacescu.dorin@umft.ro (D.N.); 5 Angiogenesis Research Center, Victor Babes University of Medicine and Pharmacy Timisoara, E. Murgu Square, No. 2, 300041 Timisoara, Romania

**Keywords:** acute pancreatitis biology, gross anatomy, pancreatic histology, inflammation and immunology, diagnostics, histopathology, biomarkers, drug discovery, epidemiology and etiology, embryology

## Abstract

Acute pancreatitis is a complex inflammatory disorder with significant morbidity and mortality. This review aims to integrate the current knowledge of pancreatic morphology and immunology with the pathogenesis of acute pancreatitis, providing a comprehensive understanding of this critical condition. We conducted an extensive literature review, synthesizing data from recent studies and authoritative sources on pancreatic anatomy, histology, immunology, and the pathophysiology of acute pancreatitis. We also incorporated epidemiological data, clinical features, diagnostic criteria, and prognostic factors. The pancreas exhibits a complex morphology with intricate interactions between its exocrine and endocrine components. Its unique immunological landscape plays a crucial role in maintaining homeostasis and orchestrating responses to pathological conditions. In acute pancreatitis, the disruption of intracellular calcium signaling leads to premature enzyme activation, triggering a cascade of events including mitochondrial dysfunction, ATP depletion, and the release of proinflammatory mediators. This process can escalate from localized inflammation to systemic complications. The interplay between pancreatic morphology, immune responses, and pathophysiological mechanisms contributes to the varied clinical presentations and outcomes observed in acute pancreatitis. Understanding the intricate relationships between pancreatic morphology, immunology, and the pathogenesis of acute pancreatitis is crucial for developing more effective diagnostic and therapeutic strategies. This integrated approach provides new insights into the complex nature of acute pancreatitis and may guide future research directions in pancreatic disorders.

## 1. Introduction

The pancreas, a vital mixed gland, digestive and endocrine, is located retroperitoneally, anterior to the bodies of the L1-L2 vertebrae, extending transversely from the duodenum on the right to the spleen on the left [1]. Its anatomical divisions—the head, neck, body, and tail—each have particular anatomical relationships and histological conformations, crucial to the gland’s functions. Thus, the pancreas serves dual purposes: the exocrine secretion of pancreatic juices from acinar cells into the duodenum via pancreatic ducts and the endocrine secretion of hormones, such as insulin and glucagon, directly into the bloodstream from the islets of Langerhans [1].

Acute pancreatitis is a complex and potentially life-threatening inflammatory disorder of the pancreas, characterized by the sudden onset of severe abdominal pain and elevated levels of pancreatic enzymes in the blood. The etiology of acute pancreatitis is diverse, with gallstone disease being the leading cause in Western countries, accounting for 20–70% of all cases [2]. Alcohol abuse is the second most common etiology, responsible for up to 30% of cases [3], followed by hypertriglyceridemia, which accounts for approximately 9% of incidents [4]. Other less frequent causes include drug-induced pancreatitis, autoimmune conditions, and various genetic predispositions [5].

According to the Atlanta classification, acute pancreatitis is broadly categorized into two types: interstitial edematous acute pancreatitis, characterized by the inflammation of the pancreatic parenchyma and surrounding tissues, and necrotizing acute pancreatitis, marked by the necrosis of the pancreatic parenchyma and peripancreatic tissues [6]. Further classification based on severity distinguishes between mild, moderately severe, and severe acute pancreatitis, with the latter involving persistent organ failure for more than 48 h [1]. Thus, this condition, characterized by acute inflammation of the pancreas, presents a spectrum of severity, ranging from mild cases, requiring only conservative management, to severe, complicated instances, associated with high morbidity and mortality. Accordingly, the mortality rate varies dramatically, from 3% in patients with mild edematous pancreatitis to as high as 20% in those with pancreatic necrosis, underscoring the critical nature of this disease [7].

Herein, acute pancreatitis represents a significant health concern, representing the leading cause of hospitalization among gastrointestinal disorders in the United States. Furthermore, the incidence of acute pancreatitis is rising globally, with reported rates of 600–700 cases/100,000 people in the United States, resulting in 200,000 to 250,000 hospital discharges annually [1,8]. This increase is partly attributed to the growing prevalence of metabolic syndrome and hypertriglyceridemia [4]. The peak incidence occurs in the fifth and sixth decades of life, affecting men and women equally, though male sex is associated with higher mortality rates [9], with mortality rates increasing with age, notwithstanding the overall decrease in mortality rates achieved in recent years [1].

The pathophysiology of acute pancreatitis is multifaceted, centered around the premature activation of digestive enzymes within the pancreas, leading to autodigestion and a cascade of inflammatory responses. This process begins with the activation of trypsinogen to trypsin within the acinar cells, rather than in the duodenal lumen. Factors such as ductal obstruction, calcium homeostasis disruption, and pH changes can trigger this premature activation [10]. The resulting tissue damage leads to the release of damage-associated molecular patterns (DAMPs), which recruit neutrophils and initiate an inflammatory cascade, promoting mitochondrial dysfunction, impaired cellular energy production, and the further release of proinflammatory cytokines [11]. These processes collectively contribute to cellular necrosis. In severe cases, this localized inflammation can progress to systemic inflammatory response syndrome (SIRS), multi-organ dysfunction syndrome (MODS), and microvascular thrombosis, which are the primary causes of morbidity and mortality in acute pancreatitis [1]. Moreover, genetic factors seemingly play a role both in the susceptibility to develop acute pancreatitis as well as its progression to chronic forms. Mutations in genes, such as *cystic fibrosis transmembrane conductance regulator (CFTR)*, *protease serine 1/cationic trypsinogen (PRSS1)*, *chymotrypsin C (CTRC)*, and *serine protease inhibitor Kazal type 1 (SPINK1)*, which are involved in trypsinogen activation and regulation, have been associated with recurrent acute pancreatitis and its evolution to chronic pancreatitis [12].

The current paper aims to provide a comprehensive analysis of pancreatic morphology and immunology, and their implications regarding the etiopathogenesis of acute pancreatitis. By integrating these theoretical aspects, alongside relevant clinical data, this review offers a broader perspective on acute pancreatitis that spans from molecular mechanisms to bedside management. This integrative approach not only enhances our understanding of the disease process but also paves the way for the development of potential novel biomarkers and therapeutic strategies in the management of acute pancreatitis. The ultimate goal is to improve early detection, enhance prognostic accuracy, and develop more effective treatments to reduce the substantial morbidity and mortality still associated with this challenging condition.

## 2. Clinical Considerations

The cornerstone of clinical management begins with accurate early diagnosis and severity assessment. Clinically, acute pancreatitis presents with a spectrum of symptoms, primarily epigastric or diffuse abdominal pain (80–95% of cases), often accompanied by nausea and vomiting (40–80%) [13]. The character of pain can offer clues to etiology; biliary causes often produce sharper pain with acute onset, while alcohol-related or metabolic causes may present more gradually with dull, generalized epigastric discomfort [1]. Additional manifestations include abdominal distension, fever, breathlessness, irritability, and altered consciousness [2]. Physical examination may reveal pyrexia, low oxygen saturation, tachypnea, tachycardia, and in severe cases, hypotension, ileus, and/or oliguria. Abdominal examination typically shows epigastric tenderness, possible guarding, and decreased bowel sounds. In extreme cases, retroperitoneal bleeding may manifest as Grey-Turner’s sign (flank ecchymosis) or Cullen’s sign (periumbilical ecchymosis) [1,2].

A comprehensive medical history is crucial, focusing on potential etiologies, such as gallstones, obesity, alcohol excess, smoking, hyperlipidemia, and pancreatitis-inducing drugs [2]. Multiple precipitating factors may coexist. Assessing alcohol use is particularly important, facilitated by tools like the 10-item Alcohol Use Disorders Identification Test (AUDIT), available in clinician-administered and self-report versions. Abbreviated AUDIT versions or alternative screening methods, including single-item questions, can also effectively identify problematic alcohol use [14].

Diagnosis relies on meeting at least two of three criteria, according to the Revised Atlanta Classification, namely, (1) characteristic abdominal pain, consistent with acute pancreatitis; (2) elevated serum lipase or amylase (at least three times the upper limit of normal); and (3) abdominal imaging findings consistent with acute pancreatitis [15].

Initial laboratory evaluation should include not only pancreatic enzymes but also a complete blood count, coagulation assays, a metabolic panel with liver function tests, serum calcium, and triglyceride levels [16]. Common signs include elevated white blood cell count, diffuse fatty necrosis on pathology, and various metabolic disturbances, such as hypocalcemia and hyperglycemia. In severe cases, patients may develop acute respiratory distress syndrome (ARDS), acute kidney injury (AKI), or disseminated intravascular coagulation (DIC). Additional tests may be necessary to determine etiology, such as genetic testing, in cases of suspected hereditary pancreatitis [17].

Abdominal ultrasound serves as the first-line imaging modality, particularly useful in identifying gallstones. Contrast-enhanced computed tomography (CT) is recommended when the diagnosis is equivocal or to assess for complications, like necrosis, typically performed 48–72 h after symptom onset. Among imaging modalities, CT is considered the gold standard, offering the detailed visualization of pancreatic enlargement, parenchymal changes, and peripancreatic fluid collections. Magnetic resonance cholangiopancreatography (MRCP) or endoscopic ultrasound (EUS) may be used for further evaluation when initial studies are inconclusive [1,18,19].

Severity assessment is crucial for guiding management and predicting outcomes. Several scoring systems are employed, including the Bedside Index for Severity in Acute Pancreatitis (BISAP), Ranson’s criteria, and the Acute Physiology and Chronic Health Evaluation II (APACHE II). Overall, the presence of persistent SIRS for ≥48 h has emerged as a strong predictor of severe acute pancreatitis [17]. The modified CT Severity Index (CTSI) provides valuable prognostic information based on imaging findings, with scores ranging from 0 to 10, correlating with increasing severity and mortality risk [20].

Treatment strategies are tailored to disease severity and etiology. Early aggressive fluid resuscitation forms the cornerstone of initial management, with Lactated Ringer’s solution being preferred, with an initial bolus of 15–20 mL/kg, followed by 3 mL/kg per hour (~250–500 mL/h), for the first 24 h, in the absence of any contraindications to fluids. Failure to respond to initial fluid resuscitation is a critical indicator of potential progression to MODS and represents grounds for a level of care upgrade. However, recent evidence suggests that overly aggressive fluid administration may lead to fluid overload without improving outcomes, emphasizing the need for careful monitoring and an individualized approach [21,22]. Pain management follows the World Health Organization (WHO) analgesic ladder, progressing from NSAIDs to opioids as needed, with recent evidence dispelling concerns about opioid-induced complications in this setting [23].

Nutritional support plays a vital role. In mild pancreatitis, early oral feeding with a soft, low-residue, low-fat diet is recommended as soon as tolerated. For severe pancreatitis or when oral intake is not feasible, nasojejunal feeding is preferred over parenteral nutrition [24]. Antibiotic use is reserved for cases of suspected infected necrosis or other infections, with prophylactic use not routinely recommended [1].

The management of specific etiologies includes early cholecystectomy for gallstone pancreatitis and endoscopic retrograde cholangiopancreatography (ERCP) within 24 h for cases complicated by cholangitis or clear biliary obstruction. In cases of hypertriglyceridemia-induced pancreatitis, the goal is to reduce triglyceride levels to below 500 mg/dL, often achieved through apheresis and insulin therapy [1,2]. The management of complications is also heterogeneous. Acute peripancreatic fluid collections often resolve spontaneously and rarely require intervention. However, pancreatic pseudocysts may require drainage if they cause symptoms or become infected. The management of pancreatic necrosis remains challenging, often requiring a step-up approach from less invasive methods (endoscopic or percutaneous drainage) to surgical debridement in refractory cases [25]. The management of organ failure and other systemic complications is crucial in severe acute pancreatitis and may require intensive care support.

Prognosis in acute pancreatitis varies widely depending on disease severity. Overall mortality has decreased in recent years to approximately 2% in most studies. However, this figure masks the significant disparity between mild and severe cases. Mild edematous pancreatitis carries a mortality rate of about 3%, while severe necrotizing pancreatitis can see mortality rates as high as 20% [26]. The BISAP score provides a quick prognostic tool at the bedside, with scores of 3–5 indicating a mortality risk greater than 15%, compared to less than 2% for scores of 0–2 [27]. As stated previously, age plays a significant role in prognosis, with increased mortality observed in older patients. The development of organ failure, particularly if persistent beyond 48 h, dramatically worsens outcomes [28]. Infected pancreatic necrosis remains a feared complication, significantly increasing morbidity and mortality. Long-term prognosis also warrants consideration. While most patients with mild acute pancreatitis recover fully, those who experience severe episodes or recurrent attacks face an increased risk of progressing to chronic pancreatitis. This progression is particularly notable in cases of alcohol-induced or genetic pancreatitis [1,2].

All in all, the management of acute pancreatitis requires a comprehensive, multidisciplinary approach tailored to individual patient factors and disease severity. The early recognition of severe cases, prompt initiation of appropriate supportive care, and timely management of complications are crucial for improving outcomes.

## 3. Morphology of the Healthy Pancreas

The pancreas is a mixed digestive gland with exocrine and endocrine secretions. Through the substances it synthesizes, the pancreas is an organ indispensable to life.

### 3.1. Embryological Development

The pancreas forms from the distal embryonic intestine, from which the dorsal and ventral buds differentiate, beginning in the 4–5th week of pregnancy. The dorsal bud forms the upper portion of the head, the entire body of the pancreas, and the tail. The ventral bud forms the lower portion of the head and the uncinate process. In the 7th week of intrauterine life, the two buds fuse. Ducts originate as cell cords that proliferate in the mesenchyme, then branch and form the lumen. The exocrine and endocrine components originate from primitive duct cells. The acini of the exocrine pancreas begin to differentiate from the 3rd month of intrauterine life and concentrate around the ducts. In acinar cells, typical zymogen granules are observed only after 20 weeks. Endocrine pancreatic cells differentiate from ductal cells in weeks 8–10 of intrauterine life. From the 3rd month, endocrine cells group to form the islets of Langerhans. After the 3rd month, pancreatic lobulation is more evident, and the stroma is reduced quantitatively [29].

Heterotopia is defined as pancreatic tissue developed outside the anatomical position of the gland. Heterotopic (ectopic) pancreatic tissue is present in 15% of people, but only 25% become functional in adults. The most common locations of heterotopia are the duodenum and stomach. Ectopic pancreatic tissue can also be observed in the jejunum, Meckel’s diverticulum, colon, and rarely, in the liver [30].

### 3.2. General Histological Structure

**Figure 1:**
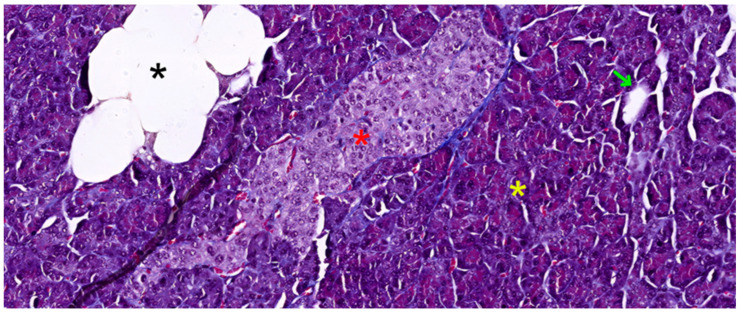
Pancreatic tissue slide in Masson trichrome stain, 200×, showing a compact islet of Langerhans (red asterisk), surrounded by serous acini (yellow asterisk), a stromal fat inclusion (black asterisk), and a small duct (green arrow).

The pancreas, a vital organ in the digestive and endocrine systems, possesses a complex structure that enables its multifaceted functions. The pancreas is a solid, encapsulated, and lobulated organ. Histologically, it consists of supporting tissue and parenchyma. The parenchyma is further divided functionally into an exocrine component, respectively an endocrine component (see Figure 1).

**Figure 2:**
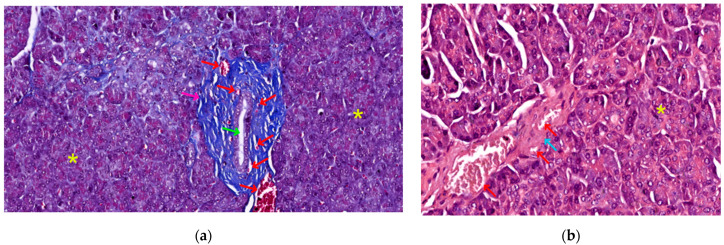
Pancreatic stromal elements: (**a**) 200×, Masson trichrome, dense irregular connective tissue trabecula (pink arrow), surrounded by serous acini (yellow asterisk), containing a wide-lumened interlobular duct (green arrow) and multiple blood vessels (red arrows) of different calibers; (**b**) 400×, HE stain, adult pancreatic stroma, comprised of loose connective tissue (blue arrow), surrounding two blood vessels (red arrows) and extending in between the serous acini (yellow asterisk).

**Figure 3:**
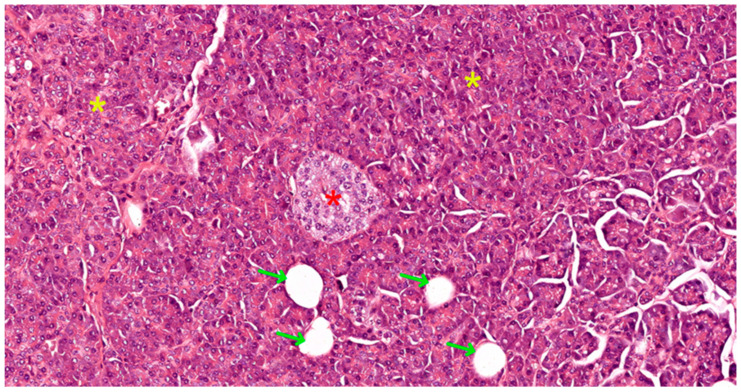
Pancreatic tissue slide in HE stain, 100×, showing a compact islet of Langerhans (red asterisk), surrounded by serous acini (yellow asterisk), and multiple small ducts (green arrows).

**Figure 4:**
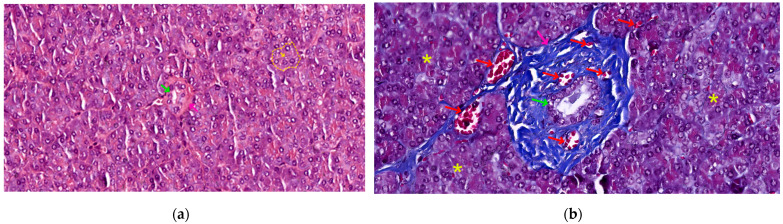
Morphological variations in pancreatic ducts: (**a**) 400×, HE stain, scant dense irregular stroma (pink arrow) surrounding a small caliber intralobular duct (green arrow), and a centro-acinar duct (yellow arrow), draining a single serous acinus (yellow outline); (**b**) 400×, Masson trichrome, rich dense irregular connective tissue trabecula (pink arrow), surrounded by serous acini (yellow asterisk), containing a large caliber intralobular duct (green arrow) and multiple blood vessels (red arrows) of different sizes.

In fact, the histological recognition criteria for the pancreas are as follows: (1) solid, encapsulated organ (connective tissue capsule); (2) lobulation present (lobules delimitated by connective trabeculae—see Figure 2a); (3) the presence of only serous acini in the lobules, as well as ducts (see Figure 1 and Figure 2a); (4) the identification in the lobule structure of pale-colored formations (islets of Langerhans) that contrast with the intensely colored appearance of serous acini (see Figure 1) [31,32].

#### 3.2.1. Supporting Connective Tissues

Its supporting tissue, or stroma, plays a crucial role in maintaining the organ’s integrity and facilitating its operations. Enveloping the pancreas is a thin capsule composed of dense irregular connective tissue. This protective layer is partially covered by peritoneum on its antero-superior face, integrating the retroperitoneal organ into the abdominal cavity. From the inner surface of this capsule, dense irregular connective tissue trabeculae extend inward. These projections, also formed of dense irregular tissue, serve multiple purposes; they define the organ’s lobular structure and house essential components, such as wide-lumened ducts, blood vessels, and nerves (see Figure 2a).

Interestingly, the quantity of stromal tissue varies with age. In adults, the stroma is notably reduced compared to that found in newborns (30% of gland volume). This adult stroma consists of loose connective tissue, rich in reticulin fibers, and contains unmyelinated nerve fibers and nerve microganglia (see Figure 2b). It also features both regular and fenestrated capillary vessels, the latter allowing for efficient molecular exchange (see Figure 2a,b). The cellular composition of the stroma includes fibroblasts and myofibroblasts, as well as a variable number of adipocytes. The quantity of these fat cells fluctuates based on an individual’s nutritional status and age (see Figure 1) [31].

#### 3.2.2. Parenchyma

The functional core of the pancreas, its parenchyma, is divided into two distinct yet interconnected components: exocrine and endocrine (see Figure 3). The exocrine component, comprising acini and ducts, is responsible for the synthesis and secretion of digestive enzymes. These enzymes are channeled into the duodenum, where they play a critical role in the breakdown of ingested nutrients.

Complementing this is the endocrine component, which includes both insular and noninsular elements. This portion of the pancreas synthesizes various hormones and releases them directly into the bloodstream. These hormones serve as key regulators of the body’s metabolism, orchestrating the intricate balance of glucose, protein, and lipid utilization throughout the organism.

This intricate arrangement of supporting tissue and functional parenchyma allows the pancreas to efficiently perform its dual role in digestion and metabolic regulation, highlighting the organ’s remarkable complexity and importance in maintaining overall physiological homeostasis.

##### The Exocrine Pancreas

The exocrine pancreas consists of acini (secretory component) and the duct system (excretory component). In the exocrine pancreas, there are exclusively serous acini that represent 85% of the pancreatic mass. The serous acini of the exocrine pancreas can have tubular shapes and can be arranged on either side of the ducts or between two ducts forming anastomotic loops (see Figure 3).

Acinar cells have a pyramidal shape, are arranged in a single row on the basement membrane, and delimit a narrow lumen that is difficult to observe in optical microscopy. The apical cytoplasm is acidophilic due to the zymogen granules it contains, while the basal cytoplasm is basophilic due to the well-developed rough endoplasmic reticulum. The secretory granules contain the following enzymes: amylase, lipase, trypsin, chymotrypsin, elastase, collagenase, and ribo- and deoxyribonuclease. With the exception of amylase, all enzymes can be useful as markers of acinar differentiation in pancreatic neoplasms [33].

**Figure 5:**
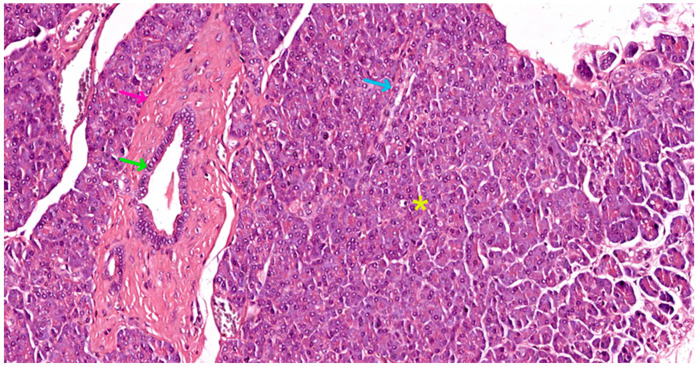
Exocrine pancreatic tissue slide in HE stain, 100×, showing a transverse section through a large interlobular duct (green arrow), enveloped within a rich dense irregular connective tissue trabecula (pink arrow), surrounded by serous acini (yellow asterisk), juxtaposed to an intralobular duct in longitudinal section (blue arrow).

The duct system is formed by:

1. Centro-acinar cells—delimiting the lumen together with acinar cells; they are small, flattened, or cuboidal in shape; the cytoplasm is pale, and the nucleus is oval; these cells are considered reserve cells for acinar and ductal cells (see Figure 4a).
2. Intralobular ducts—represented by intercalated ducts that continue the lumen of the acini and are delimited by simple cuboidal epithelium; intercalated ducts fuse to form proper intralobular ducts, which are larger and delimited by simple cuboidal or columnar epithelium; around these ducts, a loose connective stroma can be observed, with numerous reticulin fibers, yet quantitatively reduced overall; unlike major salivary glands, the exocrine pancreas does not have striated ducts (see Figure 4a,b).
3. Interlobular ducts—located in connective trabeculae; they have a wide lumen and are delimited by simple columnar epithelium; they are surrounded by well-represented, dense, irregular connective tissue, which contains fibroblasts and myofibroblasts (see Figure 5).
4. The main ducts—represented by the main Wirsung duct and the accessory Santorini duct; they are delimited by simple columnar epithelium, and in the terminal portion may also contain goblet cells [31,32,33].

##### The Endocrine Pancreas

**Figure 6:**
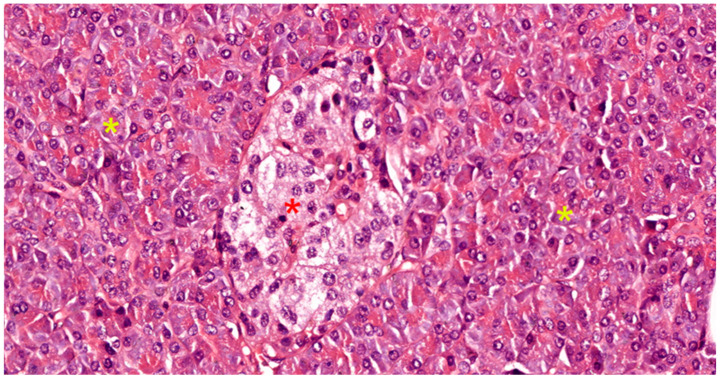
Pancreatic tissue slide in HE stain, 400×, showing a compact islet of Langerhans (red asterisk), with its various cellular morphologies, surrounded by serous acini (yellow asterisk).

The endocrine pancreas represents approximately 10% of the pancreatic mass. It consists of all hormone-secreting cells, organized either in Langerhans islets (see Figure 1, Figure 3 and Figure 6), or extraislet endocrine cell clusters, isolated among ductal and acinar cells of the exocrine pancreas, i.e., they are difficult to recognize on morphologically stained preparations, i.e., with hematoxylin–eosin (HE) or trichrome. The islet component can be either compact (regular contour) or diffuse (irregular contour).

Compact islets are located in the center of pancreatic lobules, representing 90% of all islets (see Figure 6). They are round or oval in shape, are palely colored on routine histological sections, and are well-delimited from the exocrine component. The cells of compact islets are polygonal in shape and have pale cytoplasm in routine HE staining. The nucleus is round, euchromatic, inconsistently with nucleolus, and mitoses are rare. Between cells, numerous fenestrated capillaries are present, which are difficult to observe on routinely stained preparations. Between the cells of compact islets and the exocrine component, a thin layer of connective tissue is interposed, but it does not form a proper capsule [31,32].

Regarding islet cellular phenotypes, each type of endocrine cell synthesizes only a single peptide hormone. For the identification and characterization of islet cells (see Figure 6), immunohistochemical and ultrastructural methods are used. Immunohistochemistry (IHC) has allowed the specification of islet cell distribution, using monoclonal antibodies (anti-insulin for beta cells, anti-glucagon for alpha cells, anti-somatostatin for delta cells, and anti-pancreatic polypeptide for PP cells) [32]. Thus, as discussed in Table 1, the following cell types are present in compact pancreatic islets.

Diffuse islets are located at the periphery of pancreatic lobules, representing 10% of all islets. They are large, irregular in outline, and not clearly delimited from the exocrine component. They are predominantly formed of PP cells (over 70% in diffuse islets), which secrete pancreatic peptide. The cells of diffuse islets are arranged in cords interspersed among serous acini, have a cubic or columnar shape, hyperchromatic nuclei with prominent nucleoli, and basophilic cytoplasm [32].

All pancreatic islet cells are positive for general markers of the endocrine system: chromogranin A, neuron-specific enolase, and synaptophysin.

The extraislet component consists of endocrine cells arranged in isolation among acinar and ductal epithelial cells (especially in interlobular ducts and only occasionally in intralobular ducts). Most are of the closed type, with the long axis parallel to the basement membrane. Endocrine cells are also found in the connective tissue around interlobular ducts, where they are arranged in small groups. Immunohistochemically, insulin, somatostatin, pancreatic peptide, and serotonin have been demonstrated in extraislet endocrine cells. The specific secretory products of these cells act both in a paracrine, as well as endocrine manner, some of them being released directly into the pancreatic juice [31,32,33].

### 3.3. Gross Anatomy

**Figure 7:**
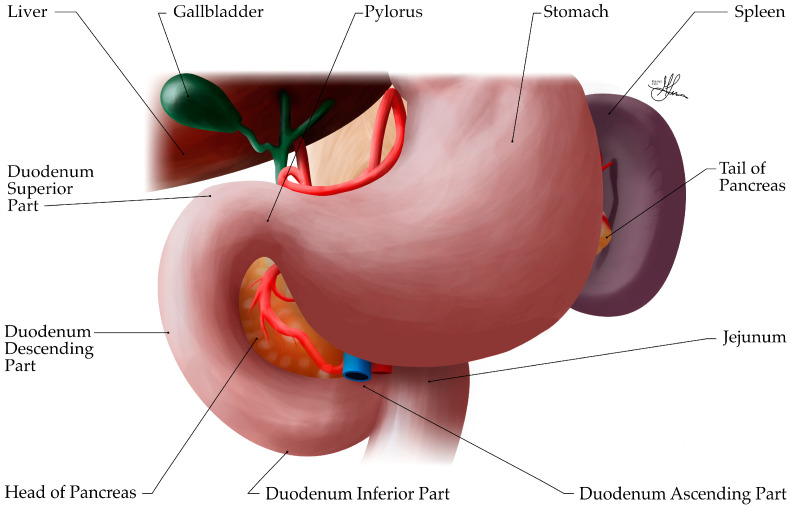
Illustration of the upper abdomen, anterior view, showing the anatomy relationships of the pancreas, i.e., covered anteriorly by the stomach, with the pancreatic head nestled within the C-shaped concavity of the duodenum and the pancreatic tail abutting the splenic hilum.

The pancreas is an elongated, soft, lobulated, mixed gland, primarily retroperitoneal, located in the upper abdomen and spanning transversely, across the posterior abdominal wall, from the concavity (C-loop) of the duodenum to the splenic hilum (see Figure 7). This passage across the midline divides the pancreas unevenly, into a larger left segment and a smaller cephalic segment, located slightly lower, across the bodies of the L1 to L3 vertebrae. Within the retroperitoneum, the pancreas is positioned at the level of the transpyloric plane (L1 vertebra), posterior to the stomach and anterior to the first and second lumbar vertebrae, with its anterior projection occupying both the epigastrium and left hypochondrium regions. In an average adult, the pancreas measures approximately 15–20 cm in length, 3–5 cm in width, and 1–2 cm in thickness, weighing about 80–120 g [34,35]. After the age of 40, the weight of the gland decreases, so that, in the 7th decade of life, it weighs approximately 70 g.

**Figure 8:**
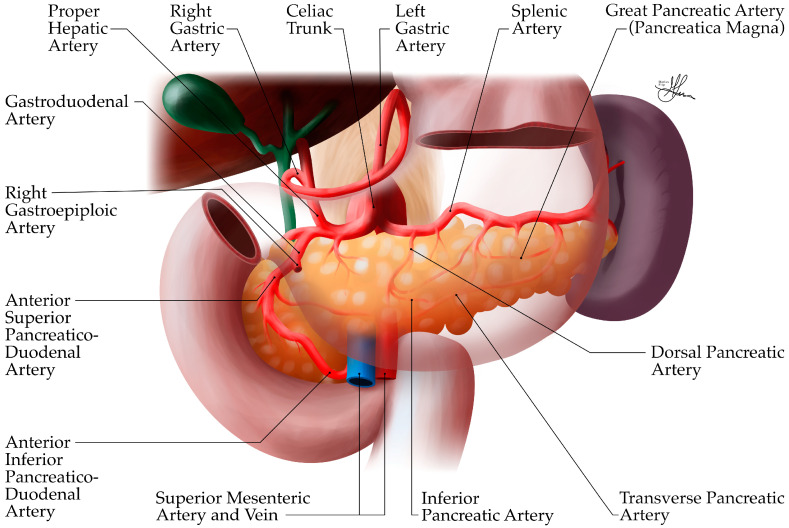
Anatomic illustration of pancreatic arterial vascularization, anterior view.

**Figure 9:**
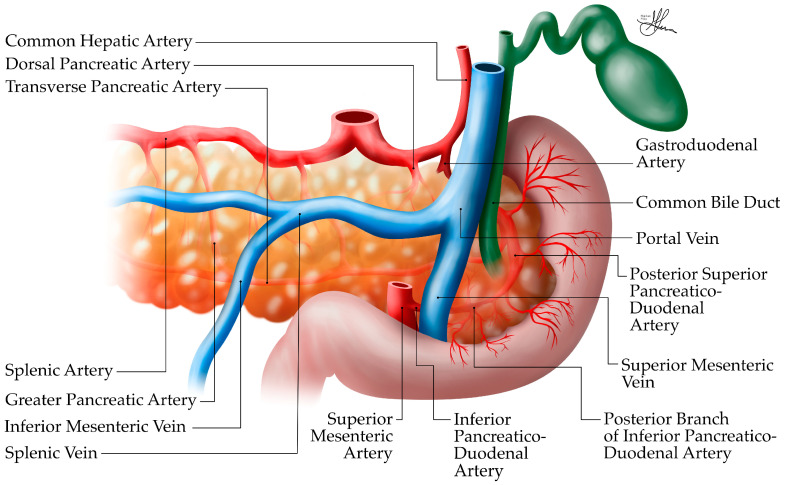
Anatomic illustration of pancreatic vascularization, posterior view.

Anatomically, the pancreas is divided into four main parts: the head, neck, body, and tail. The head is the broadest/widest part of the pancreas and lies nestled within the C-shaped concavity of the duodenum (see Figure 7). It is situated to the right of the midline at the level of the L1–L2 vertebrae and is firmly attached to the duodenum (segment II—descending part) by connective tissue, resting posteriorly on the inferior vena cava (IVC), right renal vessels, and left renal vein. The uncinate process is a superior hook-like projection, extending from the lower part of the head, curving posteriorly and medially behind the superior mesenteric vessels. It lies anterior to the aorta and IVC (see Figure 8). The neck is a short (~2 cm long), constricted portion that connects the head to the body of the pancreas, i.e., the neck is continuous with the head and merges imperceptibly into the body. It forms a groove on its posterior aspect, where it anteriorly overlies the confluence of the superior mesenteric and splenic veins, which form the portal vein (see Figure 9). The body of the pancreas represents its central portion, extending across the midline and lying posterior to the stomach, yet anterior to the aorta, splenic vein, left kidney, and left adrenal gland. The tail is the thin, leftward-ended, lateral extremity of the pancreas, that crosses the upper pole of the left kidney, as it extends towards the splenic hilum, within the splenorenal/lienorenal ligament [34,36]. Notably, this is the only intraperitoneal part of the pancreas. Herein, the splenic artery runs its parallel course towards the splenic hilum, across the superior border of the pancreas (see Figure 8).

The pancreas has important anatomical relationships with surrounding structures, which are comprehensively reviewed in Table 2. Briefly, the pancreas is related anteriorly to the stomach, from which it is separated by only the lesser sac (omental bursa). The transverse mesocolon attaches along the anterior surface of the pancreas. Posteriorly, the pancreas is related to major blood vessels including the IVC, aorta, and splenic vessels. The splenic vein courses along the posterior surface of the pancreatic body and tail (see Figure 9). The common bile duct passes behind the head of the pancreas before joining the main pancreatic duct to form the ampulla of Vater [36].

#### 3.3.1. Blood Vessels, Lymphatics, and Nerves

The pancreas is richly vascularized, but unlike other parenchymal organs, it does not have a hilum. Anastomoses between pancreaticoduodenal, dorsal pancreatic, and splenic arteries are present in most cases. Venous drainage is via the portal vein, and lymphatic drainage follows the path of blood vessels. Innervation is rich, being represented by sympathetic and parasympathetic fibers that come from the vagus nerves, celiac, and superior mesenteric nerve plexuses.

Branches from these sources ramify and enter the pancreas through connective tra-beculae. From this level, arterioles primarily irrigate the islets of Langerhans, initially peripherally (where A and D cells are located), and then centrally (where B cells are located). In this way, B cells are informed about blood glucose levels and glucagon. Between fenestrated capillaries and islet cells, there is only a very fine space, so hormone release is rapid. Fenestrated capillaries fuse and form vessels that enter the exocrine pancreas. In this way, the exocrine component is informed about hormone levels and secretes adequate amounts of enzymes. This type of vascularization has been called “cascade” [40].

##### Arterial Supply

The pancreas has a rich arterial supply (see Figure 8 and Figure 9), derived from branches of both the celiac trunk (or celiac axis) and the superior mesenteric artery (SMA), forming an extensive collateral network around the organ [36,37]. This dual blood supply is important for maintaining pancreatic viability, especially in surgical contexts.

The celiac axis, originating directly from the abdominal aorta, branches into three arteries: the splenic, left gastric, and common hepatic arteries. The common hepatic artery branches into the gastroduodenal artery (see Figure 8), which becomes the superior pancreaticoduodenal artery after passing the first part of the duodenum. This artery then splits into anterior and posterior branches. Proximal to the first jejunal arterial branches, the SMA gives rise to the inferior pancreaticoduodenal artery, which also divides into anterior and posterior branches. These superior and inferior pancreaticoduodenal branches form vascular arcades around the head of the pancreas (see Figure 8) and provide an extensive collateral network, supplying a rich blood supply to this region, i.e., the duodenum, and the head and neck of the pancreas [36]. Thus, the gastroduodenal artery, a branch of the common hepatic artery (celiac trunk), gives arterial supply to the pancreatic head, via its anterior and posterior pancreaticoduodenal branches (see Figure 8). In contrast, the inferior pancreaticoduodenal arteries, branches of the SMA, irrigate the uncinate process. Overall, the pancreatic arterial supply is quite redundant [40].

Conversely, the body and tail of the pancreas receive their arterial supply primarily from branches of the splenic artery, after it arises from the celiac trunk (see Figure 8 and Figure 9). As it courses along the superior border of the pancreas, the splenic artery gives off several important branches:

- The dorsal pancreatic artery—originating from the splenic artery, it runs posterior to the body and, finally, becomes the inferior pancreatic artery, supplying the proximal, then distal body, and tail, respectively.
- The great pancreatic artery (arteria pancreatica magna)—the largest vessel supplying the body of the pancreas from the splenic artery. Rarely, in the context of acute pancreatitis, hemorrhage occurrence has been reported at this level, a complication which can be fatal.
- The caudal pancreatic artery—supplying the tail region.
- Multiple smaller pancreatic branches—along its course [36,37,38,39,40].

However, it is worth noting that, quite frequently, significant variations in the arterial anatomy of the pancreas can be encountered (~20–30% of the population), which can impact surgical planning in pancreatic procedures [37]. Most frequently, patients may show an aberrant right hepatic artery, arising from the SMA instead of the common hepatic artery. More so, albeit rarely, the right hepatic artery may originate from the right gastric/gastroduodenal artery; whereas, the common hepatic artery may at times have an atypical origin from the SMA. Lastly, in as much as 10% of the population, the left hepatic artery may be found to aberrantly arise from the SMA, as opposed to a conventional common hepatic artery origin [38,39].

##### Venous Drainage

The venous drainage of the pancreas generally follows the arterial supply and, ultimately, drains into the portal venous system [36,37]. This is clinically significant, as it means that substances produced by the pancreas, including potential inflammatory mediators in pancreatitis, have direct access to the liver via the portal circulation. The portal vein itself is formed just posterior to the neck of the pancreas, where the splenic vein and SMV converge (see Figure 9). Unlike its arterial counterpart, the splenic vein follows a relatively straight path. It originates near the splenic hilum and closely adheres to the posterior aspect of the pancreatic tail and body. In many cases, the splenic vein is partially encased within a fibrous channel that is embedded in the pancreatic tissue for a portion of its course. The splenic vein receives between five and twelve venous tributaries draining the tail and body of the pancreas. Additionally, it accepts the inferior mesenteric vein (IMV) as a major tributary [38,39].

Venous drainage from the head and neck of the pancreas is managed by the superior and inferior pancreaticoduodenal veins. The superior pancreaticoduodenal veins have a dual drainage pattern: some empty into the right gastroepiploic vein, while others drain directly into the portal vein. The inferior pancreaticoduodenal veins, on the other hand, empty into the SMV. Despite the various routes taken by these venous channels, all venous blood from the pancreas eventually converges in the portal vein, forming a unified drainage system for the organ [36,37].

##### Lymphatic Drainage

The lymphatic system of the pancreas is complex and extensive, playing a crucial role in both normal pancreatic function and in the spread of pancreatic diseases, including cancer and inflammation [41]. The lymphatic vessels generally follow the blood vessels of the pancreas. While there is no universally accepted classification system for pancreatic lymph nodes, the more prominent system, proposed by Cubilla et al. (1978) [42], categorizes these nodes into five primary groups based on their anatomical position relative to the pancreas: superior, inferior, anterior, posterior, and splenic nodes. This regional approach has been adopted by subsequent researchers [43,44,45,46].

The superior node group receives lymphatic drainage from both anterior and posterior aspects of the pancreas’ upper region. These nodes are typically named according to their location, such as superior head nodes, superior body nodes, and gastric nodes. Some lymphatic vessels in this area may also drain into nodes within the gastro-pancreatic fold or the hepatic chain. In contrast, inferior pancreatic nodes, including the inferior head and body nodes, collect lymph from the lower portions of the pancreatic head and body. In some instances, these vessels may extend to the superior mesenteric and left latero-aortic lymph nodes [41].

Anterior nodes comprise the pyloric, anterior pancreaticoduodenal, and mesenteric lymph nodes located at the transverse colon’s mesenteric root. Their afferent lymphatic vessels form two collecting trunks that run along the anterior surface of the pancreatic head, superiorly and inferiorly, respectively. The posterior node group is dispersed similarly, inferiorly and superiorly, along the posterior surface of the cephalic pancreas. Thus, it includes the posterior pancreaticoduodenal lymph nodes, which may also receive drainage from the common bile duct and hepato-pancreatic ampulla. This group further encompasses common bile duct lymph nodes, right latero-aortic lymph nodes, and nodes near the SMA’s origin [41].

The splenic node group is responsible for draining lymph from the pancreatic tail. These nodes further connect to those at the splenic hilum, within the splenorenal/lienorenal ligament, and nodes around the pancreatic tail. They may also communicate with nodes superior or inferior to the pancreatic body.

Standring (2008) offers a simplified classification, dividing the pancreatic lymphatic network into two main regions: head/neck and body/tail [34]. The body and tail primarily drain into pancreatico-splenic nodes, with some vessels leading directly to preaortic lymph nodes. The head and neck regions have a more complex drainage system, involving nodes along the pancreaticoduodenal, superior mesenteric, and hepatic arteries, and even preaortic and celiac nodes at times. Cadaveric dissections have revealed the intricate nature of pancreatic lymphatics, particularly in the head and neck regions. However, identifying nodes along the tail and near the splenic hilum has proved challenging, highlighting the complexity of this anatomical system.

##### Innervation

The pancreas receives both sympathetic and parasympathetic innervation, which play important roles in regulating its exocrine and endocrine functions [37]. Sympathetic innervation is derived from the greater, lesser, and least splanchnic nerves, which originate from the thoracic sympathetic ganglia (T5–T12). These fibers synapse in the celiac and superior mesenteric ganglia before innervating the pancreas [34]. Parasympathetic innervation is provided by branches of the vagus nerve (cranial nerve X). These fibers travel with the arterial supply to reach the pancreas [37]. The nerve fibers form plexuses that surround the pancreatic blood vessels. In general, sympathetic stimulation inhibits pancreatic secretion, while parasympathetic stimulation enhances secretion [34].

In addition to the autonomic nervous system, the pancreas also contains an intrinsic network of neurons and nerve fibers, including peptidergic neurons. These neurons can secrete various neurotransmitters and neuropeptides, such as somatostatin, vasoactive intestinal peptide (VIP), calcitonin-gene-related peptide (CGRP), and galanin, that influence both exocrine and endocrine pancreatic functions [37].

The complex innervation of the pancreas is not only important for its normal physiological function but also plays a role in pain perception in pancreatic diseases. The rich nerve supply of the pancreas contributes to the severe pain often associated with conditions like acute pancreatitis.

#### 3.3.2. Ductal System Topography

The pancreatic ductal system is crucial for collecting and delivering pancreatic exocrine secretions to the duodenum. It consists of the main pancreatic duct, the accessory pancreatic duct, and a complex network of smaller ducts (discussed in the previous Section 3.2.2).

**Figure 10:**
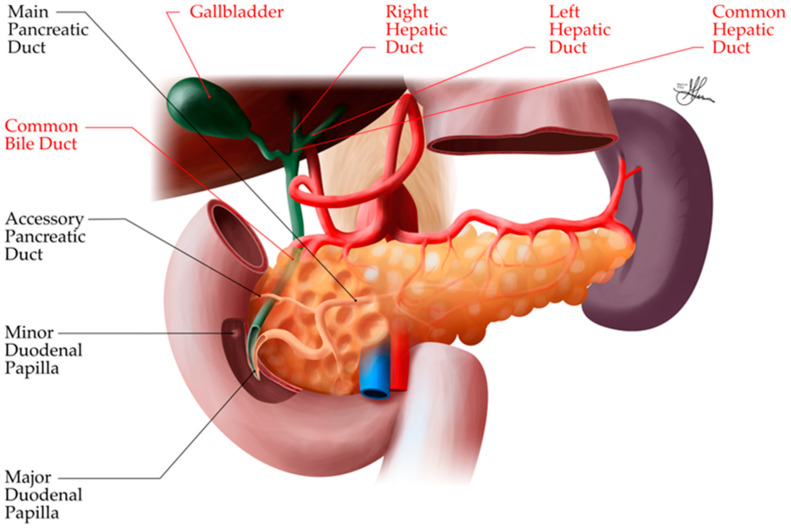
Illustration of dissected pancreas, anterior view, showing the anatomy of the pancreatic ductal system.

The main pancreatic duct (duct of Wirsung) typically measures 2–3 mm in diameter and runs the length of the pancreas from tail to head (see Figure 10). It begins in the tail and courses through the body, gradually increasing in diameter as it receives drainage from smaller branch ducts. In the head, it turns inferiorly and posteriorly to join the common bile duct, forming the hepato-pancreatic ampulla (ampulla of Vater) [38,39,40].

The main pancreatic duct and common bile duct usually enter the duodenum together at the major duodenal papilla, located on the posteromedial wall of the second part of the duodenum, about 7–10 cm distal to the pylorus. The sphincter of Oddi, composed of smooth muscle fibers, surrounds the ducts at their junction and regulates the flow of pancreatic and biliary secretions into the duodenum [40].

In 60–70% of individuals, the pancreatic and bile ducts unite within the duodenal wall to form a common channel (1–5 mm long) before opening at the papilla. In the remaining cases, the ducts may open separately or have a very short common channel.

The accessory pancreatic duct (duct of Santorini) is a smaller duct that typically drains the anterior portion of the pancreatic head. It usually communicates with the main pancreatic duct and opens into the duodenum at the minor duodenal papilla, located about 2 cm proximal to the major papilla. The accessory duct may be patent, stenotic, or obliterated [40].

This extensive ductal network ensures the efficient collection and transport of pancreatic enzymes and bicarbonate-rich fluid from the acinar and ductal cells to the duodenum.

##### Anatomical Variations and Anomalies

Variability during the embryological fusion process of the dorsal and ventral pancreas can lead to multiple congenital variants of the pancreatic ducts. Several anatomical variations and congenital anomalies of the pancreatic ductal system have been described [38,39]:

1. Pancreas divisum: the most common congenital variant (5–10% of the population), where the dorsal and ventral pancreatic ducts fail to fuse during development.
2. Annular pancreas: a rare anomaly where pancreatic tissue encircles the duodenum, potentially causing duodenal obstruction.
3. Ansa pancreatica: an anatomical variant where the main pancreatic duct forms a loop configuration within the pancreatic head.
4. Santorinicele: a cystic dilatation of the terminal portion of the accessory pancreatic duct.
5. Anomalous pancreatico-biliary junction: a condition where the pancreatic and bile ducts join outside the duodenal wall, forming an abnormally long common channel.

Understanding these anatomical variations is crucial for interpreting imaging studies, planning surgical interventions, and managing pancreatic diseases. The complex anatomy of the pancreatic ductal system, including its variations and potential anomalies, plays a significant role in the pathophysiology of various pancreatic diseases, including acute pancreatitis.

## 4. Immune Landscape of the Pancreas—Pathological Implications

The pancreas, a vital organ with dual endocrine and exocrine functions, exhibits a complex and dynamic relationship with the immune system. This intricate interplay is crucial for maintaining homeostasis and orchestrating responses to pathological conditions. Histologically, the pancreas is enveloped by a thin layer of loose connective tissue, which extends into the glandular parenchyma, subdividing it into numerous lobules. The parenchyma primarily consists of acini, ducts, and islet cells, interspersed with small vascular structures, lymphatics, and nerve tissue. This organization facilitates close contact between pancreatic cells and immune components, creating a microenvironment conducive to intimate interactions between stromal and invading immune cells and the pancreatic parenchyma [40].

Moreover, the pancreatic lymphatic system is characterized by an imperfect tripartite drainage pattern, closely aligned with its anatomical regions: (1) the pancreatic head drains mainly through the pancreaticoduodenal lymph nodes; (2) the pancreatic body’s lymph flows mainly through the superior and inferior pancreatic lymph nodes; and (3) the pancreatic tail drains via the splenic hilar lymph nodes. Ultimately, these lymphatic pathways converge towards the celiac and superior mesenteric lymph nodes, forming a comprehensive network that enables bidirectional immune cell trafficking and facilitates systemic immune responses.

As we delve into the realm of pancreatic inflammation, the innate immune system takes center stage. Macrophages and neutrophils, the frontline defenders of innate immunity, play pivotal roles in the inflammatory processes of the pancreas. These cells exhibit remarkable plasticity, capable of dramatically altering their phenotype in response to environmental cues. In the context of pancreatic inflammation, they upregulate the expression of key adhesion molecules, most notably P-selectin and intercellular adhesion molecule 1 (ICAM-1). This increased expression facilitates the recruitment and adhesion of additional immune cells to sites of inflammation within the pancreas [40].

Moreover, these activated innate immune cells become potent secretors of inflammatory mediators. They release macrophage inflammatory protein 2 (MIP-2), a powerful chemokine that further amplifies the inflammatory response by attracting more neutrophils to the site. Additionally, they produce a cocktail of reactive oxygen species, which, while intended to combat potential pathogens, can also cause significant collateral damage to pancreatic tissues. The release of elastase and matrix metalloproteinase 9 by these cells further contributes to tissue remodeling and potential damage [40].

Recent research has shed light on a fascinating aspect of neutrophil function in acute pancreatitis: the formation of neutrophil extracellular traps (NETs). These web-like structures, composed of extracellular deoxyribonucleic acid (DNA), histones, and neutrophil-derived granule proteins, are abundantly formed in pancreatic tissue during acute inflammation. NETs primarily drive tissue injury through multiple mechanisms: they directly activate trypsinogen in acinar cells through the signal transducer and activator of the transcription 3 (STAT3) pathway; they promote local inflammation by increasing MIP-2/CXCL2 production and neutrophil recruitment; and their histone components exhibit direct cytotoxicity to acinar cells. NETs also contribute to systemic complications by elevating proinflammatory mediators, such as interleukin (IL)-6 and high-mobility groups protein 1 (HMGB1), while promoting lung injury. The pathogenic role is supported by evidence that NET degradation with DNase I significantly reduces pancreatic damage, neutrophil infiltration, and systemic inflammation in experimental models [47]. However, in the specific context of infected pancreatic necrosis, NETs can serve limited protective functions by forming temporary barriers between necrotic and viable tissue areas and helping eliminate pathogens in infected regions. This duality illustrates the context-dependent nature of NET function, though their overall impact in acute pancreatitis remains predominantly harmful [47,48]. Notably, elevated levels of NET components in plasma correlate with disease severity in patients, suggesting their potential as both therapeutic targets and biomarkers [47].

The progression of acute pancreatitis offers a stark illustration of how local pancreatic inflammation can rapidly escalate into a systemic crisis. The process begins with the premature activation of pancreatic enzymes within the organ itself. This activation triggers a cascade of events, including the induction of autophagy in acinar cells. As these cells succumb to damage, they release a plethora of proinflammatory mediators into the local environment. This release acts as a clarion call to the immune system, rapidly recruiting leukocytes to the site of inflammation.

As leukocytes infiltrate the pancreatic tissue, they further amplify the inflammatory response. They increase the expression of adhesion molecules on their surfaces and on the endothelial cells lining pancreatic blood vessels. This upregulation facilitates the even greater infiltration of immune cells from the circulation into the pancreatic tissue. The infiltrating cells, now fully activated, produce an array of cytokines and other inflammatory mediators, creating a self-perpetuating cycle of inflammation.

The consequences of this unchecked inflammation are severe. Vascular permeability increases dramatically, leading to the development of edema around the inflamed pancreatic tissue. The local microcirculation becomes disorganized, resulting in areas of tissue hypoxia. This oxygen deprivation, combined with the direct damaging effects of inflammatory mediators, can lead to substantial tissue necrosis. In severe cases, the local pancreatic inflammation can spiral into a systemic inflammatory response, potentially culminating in MODS and, in the most severe cases, death.

At the molecular level, several signaling pathways play crucial roles in orchestrating and amplifying the inflammatory response in pancreatic tissue. The toll-like receptor 4 (TLR4) pathway is particularly noteworthy. TLR4, a pattern recognition receptor, can be activated by both exogenous factors, like lipopolysaccharide from Gram-negative bacteria, and endogenous danger signals released from damaged pancreatic cells. Once activated, TLR4 initiates a signaling cascade through the MyD88-dependent pathway, ultimately leading to the activation of IκB kinase [48].

Simultaneously, endogenous proinflammatory factors, such as tumor necrosis factor-α (TNF-α) can bind to their specific receptors (TNFR or IL-1R), activating IκB kinase through a parallel, MyD88-independent pathway. Both of these pathways converge on a critical point: the activation of nuclear factor kappa B (NF-κB). Once activated, NF-κB translocates to the nucleus, where it binds to the promoter regions of numerous proinflammatory genes. This binding results in the increased transcription of a wide array of inflammatory mediators, including more TNF-α, various adhesion molecules, and chemokines.

The role of platelet-activating factor (PAF) in this process cannot be overstated. Released from activated platelets, PAF significantly increases vascular permeability and promotes the infiltration of inflammatory cells. It acts synergistically with other inflammatory mediators to amplify the overall inflammatory response, creating a perfect storm of inflammation within the pancreatic tissue [48].

While the innate immune response is rapid and robust, the adaptive immune system also plays a critical role in pancreatic pathology, particularly in chronic conditions and malignancies. In the context of pancreatic cancer, for instance, there is a marked dysregulation of T cell populations. CD4+ and CD8+ T cells, typically the workhorses of the adaptive immune response, are often significantly downregulated in the tumor microenvironment. This downregulation is accompanied by an upregulation of regulatory T cells (Tregs), which can suppress the antitumor immune response [49].

Furthermore, the function of cytotoxic T lymphocytes (CTLs) and natural killer (NK) cells, both crucial for tumor surveillance and elimination, is often impaired in pancreatic cancer. This immunological imbalance creates an environment conducive to tumor growth and metastasis, highlighting the critical role of the immune system in both protecting against and, when dysregulated, potentially promoting pancreatic pathology [49].

The intricate interplay between the pancreas and the immune system has significant clinical implications. In recent years, there has been growing interest in immunomodulatory therapies for pancreatic disorders. In the context of acute pancreatitis, for example, treatments targeting specific immune pathways have shown promise in mitigating the severity of the disease. These approaches range from blocking specific proinflammatory cytokines to modulating the function of particular immune cell subsets.

Looking to the future, several key areas of research hold promise for advancing our understanding and treatment of pancreatic disorders. Elucidating the precise mechanisms of NET formation and function in pancreatic inflammation could lead to novel therapeutic approaches for managing acute pancreatitis [47]. In the field of pancreatic cancer, developing strategies to effectively modulate the tumor microenvironment and enhance antitumor immunity remains a critical goal.

Additionally, there is growing interest in leveraging our understanding of pancreatic immunology for diagnostic purposes. The development of biomarkers based on immune cell profiles or circulating inflammatory mediators could potentially allow for earlier detection of pancreatic diseases, including cancer, where early diagnosis is crucial for improving outcomes.

Overall, the immunology of pancreatic tissues represents a frontier of medical research, characterized by complex interactions between diverse cell types and molecular pathways. The pancreas exists in a delicate immunological equilibrium, with its unique anatomy and physiology creating a microenvironment where immune cells and pancreatic tissues are in constant communication. This dynamic interplay not only maintains normal function but also plays a critical role in the pathogenesis of pancreatic diseases. As our understanding of these intricate mechanisms continues to grow, so too does the potential for developing novel, targeted therapies for a range of pancreatic disorders, from inflammatory conditions to malignancies. The future of pancreatic medicine lies in unraveling these complex immunological tapestries, promising new hope for patients suffering from these challenging diseases.

## 5. Histopathology of Acute Pancreatitis

Central to exocrine pancreas pathology is acute pancreatitis, an extremely serious, life-threatening condition, manifesting a strong parenchymal inflammatory response caused by the release of active pancreatic enzymes that digest the pancreatic tissues (i.e., a process of autodigestion occurs). Clinically, it manifests with characteristic sudden-onset upper abdominal pain, abdominal distension, acute surgical abdomen, hypotension, and/or hemorrhagic shock. Biologically, pancreatic enzymes will be elevated in the blood. Histologically, it represents a spectrum of tissue damage patterns that correlate with the severity and etiology of the disease. Microscopically, areas of necrosis, hemorrhagic suffusions with hemosiderin deposits, are observed in the pancreas; whereas, the prognosis of the condition is very poor. Understanding the histopathological features is crucial for accurate diagnosis, assessment of disease progression, and guiding clinical management.

At its core, acute pancreatitis involves functional and/or structural acinar cell damage, rarely accompanied by duct cell necrosis. The initial insult is typically noninfectious; although, infectious agents can occasionally be implicated. Pathologically, acute pancreatitis can be broadly categorized into two forms: acute interstitial edematous pancreatitis, affecting 90–95% of patients, and acute necrotizing pancreatitis, seen in 5–10% of cases [6,15,50]. The milder interstitial form is frequently associated with gallstone disease, while the more severe necrotizing form often has an alcoholic etiology.

The histopathological patterns of tissue necrosis in acute pancreatitis can be classified into three distinct types. Type 1 necrosis, the most common pattern, primarily affects the fatty tissue in interstitial spaces within the pancreas and peripancreatic areas. Type 2 necrosis involves the duct epithelium, while Type 3 necrosis is characterized by acinar cell destruction [50]. These patterns provide insights into the underlying pathophysiological mechanisms and can guide clinical management strategies.

**Figure 11:**
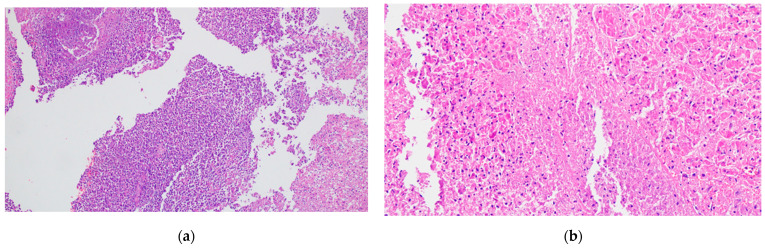
Acute pancreatitis tissue samples, stained in hematoxylin–eosin (HE): (**a**) 100×, acute inflammation; (**b**) 200×, coagulative necrosis of pancreatic tissue (details).

In acute interstitial edematous pancreatitis, the pancreas exhibits edematous swelling with scattered, tiny, white spots of fatty tissue necrosis on its surface and within interlobular spaces. Microscopically, the interstitial fibrous tissue, adipose tissue, and pancreatic parenchyma appear edematous and infiltrated by acute inflammatory cells, predominantly neutrophils (see Figure 11a). Limited fat necrosis may be observed. As the disease progresses, macrophages and lymphocytes gradually replace the neutrophils [50,51].

**Figure 12:**
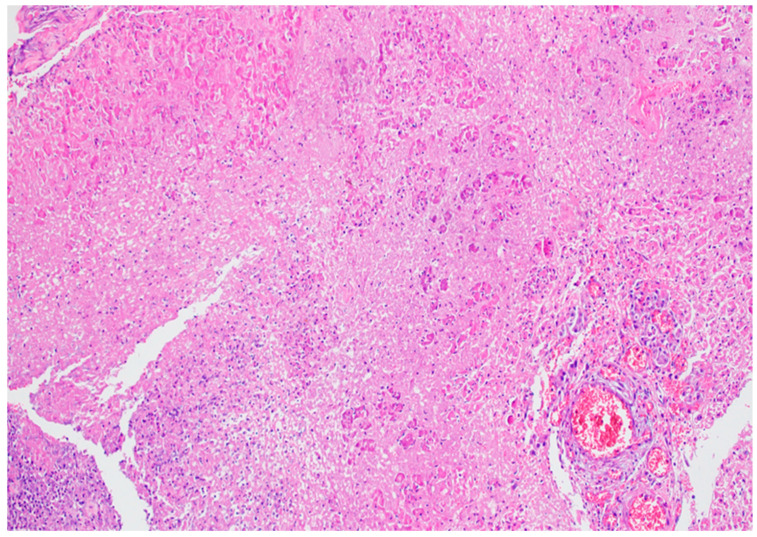
Acute pancreatitis tissue sample, stained hematoxylin–eosin (HE), 200×, showing vascular hyperemia, coagulative necrosis of pancreatic tissue, and inflammatory infiltrate.

Acute necrotizing pancreatitis presents a more severe histological picture [50]. Grossly, large confluent areas of white fat necrosis are evident in the peripancreatic tissue. The pancreatic parenchyma shows extensive necrosis, often less pronounced than the peripancreatic changes. The extent of necrosis may correlate with the amount of interlobular fatty tissue, potentially explaining the increased severity in obese individuals. Microscopically, there is marked hemorrhage, panlobular coagulative necrosis (see Figure 11b), and widespread fat necrosis extending into peripancreatic tissues. Intravascular thrombi are frequently observed, contributing to the ischemic damage (see Figure 12).

The evolution of histopathological changes in acute pancreatitis follows a temporal pattern. In the initial phase of interstitial pancreatitis, edema rich in pancreatic enzymes is usually resolved by macrophages within days, leaving minimal residual changes. In necrotizing pancreatitis, large areas of fat necrosis undergo liquefaction. Smaller foci (2–5 cm) may be reabsorbed, while larger necrotic areas (>5 cm) often persist. These persistent areas become surrounded by macrophages and inflammatory cells, forming a thin layer of granulation tissue within 10–20 days of disease onset. This histological picture corresponds to the radiologically described “acute necrotic collection” [52].

As the disease progresses beyond 20–30 days, the granulation tissue is gradually replaced by fibrosis containing collagen types 1 and 3, forming a well-defined wall. This advanced stage correlates with the radiological term “walled-off necrosis,” previously known as “pseudocyst associated with necrosis” [53]. These lesions are typically found outside the pancreas, particularly around the pancreatic head [52]. The presence of amylase within these structures suggests communication with the pancreatic duct system, which may lead to complications, such as compression or the erosion of adjacent structures.

The infection of necrotic tissue by bacteria or fungi, usually originating from the gut, can occur during the early stages of demarcation (days 4–20). This complication significantly impacts patient prognosis and requires prompt clinical intervention.

The resolution of pancreatic necrosis often results in interlobular fibrosis replacing the damaged tissue [53]. In cases of recurrent attacks of necrotizing pancreatitis, this necrosis–fibrosis sequence can involve large interlobular ducts and the main pancreatic duct, potentially leading to the development of chronic pancreatitis.

Acute pancreatitis with a type 2 necrosis pattern, characterized by the disseminated ductal necrosis of small-to-medium-sized interlobular ducts, is less common. It is often associated with prolonged circulatory failure or certain genetic conditions, like hereditary pancreatitis. The affected ducts contain granulocytes mixed with eosinophilic secretions, leading to duct rupture and periductal necrosis. This pattern may result in long-term structural changes, such as irregular duct dilatations and periductal scarring [50].

The type 3 necrosis pattern, featuring scattered intralobular foci of acinar cell necrosis without significant fat or ductal involvement, is indicative of infectious etiology. This pattern is often associated with viral or bacterial agents and typically results in a milder clinical course [50].

The histopathological features of acute pancreatitis correlate with the radiological and clinical criteria outlined in the 2012 Atlanta Classification [15]. Interstitial edematous pancreatitis corresponds to mild clinical presentation with minimal complications. Moderately severe cases may show peripancreatic fat necrosis and pseudocyst formation. Severe necrotizing pancreatitis is characterized by extensive pancreatic and peripancreatic necrosis, often with hemorrhage, and may progress to walled-off necrosis [54].

Understanding the histopathological patterns in acute pancreatitis is crucial for elucidating disease mechanisms, guiding clinical management, and predicting outcomes. However, several questions remain unanswered, particularly regarding the precise pathophysiological mechanisms governing disease severity and progression. Future research focusing on the correlation between histopathological findings and emerging molecular markers may provide further insights into this complex and potentially life-threatening condition.

## 6. Etiology and Pathogenesis of Acute Pancreatitis

Acute pancreatitis is a multifactorial condition with the most common causes being gallstones, alcohol use [1], and hypertriglyceridemia [55], accounting for a significant proportion of cases globally. However, the incidence of each etiology varies based on geographic region and socioeconomic factors [56,57,58]. In the United States, biliary disease and alcohol use are predominant, representing approximately 40% and 35% of cases, respectively [59,60]. Gallstone pancreatitis occurs when a stone obstructs the bile duct or sphincter of Oddi, leading to injury of the pancreatic acinar cells due to increased ductal pressures [61]. The anatomical variations in the bile and pancreatic ducts may influence the risk and severity of this condition.

Forensic pathology commonly encounters acute pancreatitis in cases involving alcohol use, either alone or superimposed on chronic pancreatitis, due to the inherent selection bias of alcohol-related deaths falling under its jurisdiction [62]. Alcohol exerts its damaging effects through several mechanisms, such as causing intracellular accumulation of digestive enzymes, premature activation and secretion, the increased permeability of pancreatic ductules, and promoting protein plugs that block duct outflow [62]. Another relatively frequent cause is hypertriglyceridemia, which leads to the formation of toxic-free fatty acids, further contributing to pancreatic injury [55].

Other less common etiologies of acute pancreatitis are diverse and include drug-induced pancreatitis, idiopathic origins, and post-procedural factors, such as ERCP or abdominal surgeries. Additionally, autoimmune pancreatitis (type I, systemic IgG4 disease-related, and type II), viral infections (coxsackie, cytomegalovirus, Epstein–Barr virus, hepatitis A/B/C, HIV, mumps, rubella, and varicella), bacterial infections (e.g., Mycoplasma, Mycobacterium tuberculosis), trauma, smoking, congenital anomalies (e.g., annular pancreas), and various toxins (e.g., scorpion or snake venom, organophosphate poisoning) have all been implicated [2], as seen in Table 3.

Hypercalcemia, parasitic infections, genetic predispositions (e.g., *CFTR* gene mutations, *PRSS1* gain-of-function mutations), and conditions, such as vasculitis (e.g., systemic lupus erythematosus), also play a role. Renal disease and autoimmune disorders contribute to the complexity of this condition [1,2].

The central pathophysiological event in acute pancreatitis is the premature activation of trypsinogen to trypsin within the pancreatic acinar cells, instead of within the duct lumen. This leads to the activation of additional digestive enzymes, such as elastase and phospholipases, which contribute to extensive tissue destruction. Factors, such as increased ductal pressure, disturbances in calcium homeostasis, and changes in pH, can precipitate this premature activation (see Table 4). Many toxins causing pancreatitis, such as alcohol, contribute to ATP depletion, resulting in elevated intra-acinar calcium concentrations that trigger the early activation of trypsinogen [1,2]. This cascade initiates the release of DAMPs, which are pivotal in triggering the recruitment of neutrophils and the subsequent inflammatory response. As inflammation intensifies, systemic manifestations develop, often leading to SIRS, MODS, and microvascular thrombosis. The latter contributes to complications, such as DIC, shock, and capillary permeability, which are critical determinants of morbidity and mortality in acute pancreatitis [15,54].

Genetic predispositions play a role in recurrent and chronic pancreatitis (see Table 4). Genes, such as *CFTR*, *PRSS1*, and *SPINK1*, are involved in trypsinogen activation and regulation, with mutations contributing to recurrent acute episodes and progression to chronic pancreatitis. This genetic link highlights the spectrum from acute-to-chronic forms of the disease and underscores the importance of genetic factors in disease pathogenesis [1,2,10].

## 7. Conclusions and Future Research Directions

This comprehensive review of pancreatic morphology, immunology, and the pathogenesis of acute pancreatitis underscores the complex interplay between structure, function, and disease processes within the pancreas. By integrating our understanding of pancreatic anatomy and histology with recent advances in pancreatic immunology and the molecular mechanisms of acute pancreatitis, we gain a more nuanced perspective on this challenging clinical entity.

The intricate architecture of the pancreas, with its closely integrated exocrine and endocrine components, provides the structural basis for its vital physiological functions. However, this same complexity also underlies its vulnerability to inflammatory processes. The emerging field of pancreatic immunology reveals a delicate balance between immune surveillance and tolerance, which, when disrupted, can precipitate or exacerbate pancreatic inflammation.

In acute pancreatitis, the convergence of various etiological factors on disrupted calcium signaling and premature enzyme activation highlights common pathways that may be targeted for therapeutic intervention. The progression from localized inflammation to systemic complications emphasizes the need for early recognition and intervention in the disease process.

The epidemiological data presented underscores the significant burden of acute pancreatitis, with its substantial incidence and potentially high mortality rates, particularly in severe cases. The predominance of gallstones, alcohol, and hypertriglyceridemia as leading causes points to the importance of preventive strategies and lifestyle modifications in reducing disease incidence.

Advances in diagnostic imaging, particularly CT scanning, have greatly enhanced our ability to assess the extent and severity of pancreatic inflammation. However, the challenge remains in developing more sensitive and specific early markers of disease severity to guide management decisions.

Looking forward, several key areas warrant further investigation:

1. The elucidation of the precise mechanisms by which different etiological factors converge on common pathophysiological pathways in acute pancreatitis.
2. The development of targeted therapies that can interrupt the inflammatory cascade early in the disease process.
3. The identification of genetic and environmental factors that predispose individuals to severe or recurrent pancreatitis.
4. The exploration of the long-term consequences of acute pancreatitis, including the potential progression to chronic pancreatitis or pancreatic cancer.
5. The investigation of the role of the pancreatic microbiome in health and disease.

In conclusion, our integrated understanding of pancreatic morphology, immunology, and the pathogenesis of acute pancreatitis provides a solid foundation for future research and clinical innovation. By continuing to bridge the gap between basic science and clinical practice, we can aspire to develop more effective strategies for the prevention, early detection, and treatment of acute pancreatitis, ultimately improving outcomes for patients affected by this significant health challenge.

## Figures and Tables

**Table 1:** Characterization of cellular subtypes in compact pancreatic Langerhans islets [31,32].

Type	% ^1^	Location in Compact Islets	Ultrastructure	Secretion
B (beta)	60–70%	Center	Granules with crystalloid	Insulin
A (alpha)	15–20%	Periphery	Eccentric electron-dense material	Glucagon
D (delta)	10–15%	Periphery	Small, uniformly electron-dense granules	Somatostatin
PP	5%	Nonspecific	Granules with variable electron density or homogeneously electron-dense (depending on area)	Pancreatic polypeptide

^1^ Represents the percentage of cells belonging to a specific subtype out of the total cellular population of compact pancreatic Langerhans islets.

**Table 2:** Topographical review of the anatomical relationships of the pancreas [34,36,37,38,39,40].

Aspect ^1^	Organ	Relationship
**Anterior**	Stomach	The posterior wall of the stomach is separated from the anterior surface of the pancreas by only the lesser sac (omental bursa).
	Transverse colon/mesocolon	An important surgical landmark, the transverse mesocolon attaches to the anterior pancreatic surface.
	Small intestine	Loops of jejunum may lie anterior to the pancreas, especially its lower part.
**Posterior**	Aorta	The body of the pancreas crosses anterior to the aorta, at the level of the celiac trunk and superior mesenteric artery (SMA) origins.
	Inferior vena cava (IVC)	Lies posterior to the head of the pancreas.
	Left kidney/adrenal gland	Posterior to the body and tail of the pancreas.
	Splenic vein	Courses along the posterior surface of the pancreatic body and tail.
	Left renal vein	Passes behind the neck and body of the pancreas
	Vertebral column	The pancreas lies anterior to the L1 and L2 vertebrae.
**Lateral**	Spleen	The tail of the pancreas extends towards the splenic hilum within the splenorenal/lienorenal ligament.
	Splenic artery	Courses along the superior border of the pancreas laterally.
**Medial**	Duodenum	The C-shaped duodenal loop wraps around the head of the pancreas. The second part of the duodenum lies lateral to the pancreatic head and contains the major duodenal papilla (ampulla of Vater), where the pancreatic duct and common bile duct typically enter the duodenum.
	Superior mesenteric vessels	The superior mesenteric artery and vein pass behind the neck of the pancreas and in front of the uncinate process.
**Superior**	Celiac trunk	Originates from the aorta just above the superior border of the pancreas.
	Common hepatic artery	Often courses along the superior border of the pancreas before turning upwards towards the liver.
**Inferior**	Duodenojejunal flexure	Lies inferior to the body of the pancreas.
	Left colic flexure	May come into contact with the inferior border of the pancreatic tail.

^1^ Represents the anatomical aspect of the pancreas being discussed, i.e., anterior/posterior, lateral/medial and superior/inferior, respectively.

**Table 3:** Etiology of acute pancreatitis [1,2,55,56,57,58,59,60,61,62].

More Common Causes	Less Common Causes
Gallstones Alcohol use Hypertriglyceridemia Drug-induced pancreatitis Post-endoscopic retrograde cholangiopancreatography (ERCP) Idiopathic Ampullary stenosis (or sphincter of Oddi dysfunction type I) Trauma Smoking Toxins (e.g., scorpion venom, organophosphate poisoning) Renal disease (e.g., hemodialysis)	Malignancy/tumor Autoimmune pancreatitis (Type I and II) Hyperparathyroidism Hypercalcemia Viral infections (e.g., coxsackie, cytomegalovirus, echovirus, Epstein–Barr virus, hepatitis A/B/C, HIV, mumps, rubella, and varicella) Bacterial infections (e.g., Campylobacter jejuni, Legionella, Leptospirosis, Mycobacterium avium, Mycobacterium tuberculosis, and Mycoplasma) Genetic disorders (e.g., hereditary pancreatitis, cystic fibrosis, α1-antitrypsin deficiency) Parasitic infections (Ascaris lumbricoides, Cryptosporidium, Clonorchis sinensis, Microsporidia) Congenital anomalies (e.g., annular pancreas) Vasculitis (e.g., polyarteritis nodosa, systemic lupus erythematosus)

**Table 4:** Systematic summary of the molecular mechanisms involved in the pathogenesis of acute pancreatitis, their biological implications, and the derived potential targeted therapeutic interventions [63,64,65,66,67,68,69,70,71,72,73,74,75,76,77,78,79,80,81,82,83,84,85,86,87,88,89,90,91,92,93,94,95,96,97,98,99,100,101,102,103,104,105,106].

Mechanisms	Main Components	Key Players	Detailed Process	Consequences	Interactions	Potential Interventions
**1. Premature Enzyme Activation [67,68,69,70]**	Trypsinogen Autoactivation [67]	- Cationic trypsinogen (*PRSS1*) - Anionic trypsinogen (*PRSS2*) - Trypsin	- pH drop in acinar cells - Conformational change in trypsinogen - Autocatalytic cleavage of trypsinogen activation peptide - Formation of active trypsin	- Initiates zymogen activation cascade - Damages cellular structures - Triggers inflammatory responses	- Amplifies calcium signaling disruption - Activates NF-κB pathway - Induces acinar cell injury	- Trypsin-specific inhibitors - pH modulators - Trypsinogen stabilizers
	Cathepsin B-Mediated Activation [68]	- Cathepsin B - Trypsinogen - Lysosomal membrane proteins	- Lysosomal membrane permeabilization - Release of cathepsin B into cytosol - Cathepsin B cleaves trypsinogen activation peptide - Formation of active trypsin	- Accelerates trypsinogen activation - Contributes to lysosomal dysfunction - Enhances cellular damage	- Interacts with autophagy pathways - Contributes to oxidative stress - Amplifies inflammatory cascade	- Cathepsin B inhibitors (e.g., CA-074Me) - Lysosomal membrane stabilizers - Autophagy modulators
	Impaired Protective Mechanisms [69]	- SPINK1 - CTRC - α1-antitrypsin	- Overwhelmed SPINK1 capacity - Reduced CTRC-mediated trypsin degradation - Insufficient α1-antitrypsin levels	- Unchecked trypsin activity - Prolonged enzyme activation - Extended tissue damage	- Affects ER stress responses - Modulates inflammatory intensity - Influences cell death pathways	- SPINK1 analogues - CTRC activators - Recombinant α1-antitrypsin therapy
	Zymogen Co-localization [70]	- Zymogen granules -Lysosomes - Vacuoles	- Formation of large vacuoles - Fusion of zymogen granules with lysosomes - Creation of environment for enzyme activation	- Facilitates enzyme activation - Disrupts normal cellular architecture - Contributes to organelle dysfunction	- Linked to autophagy impairment - Affects intracellular trafficking - Contributes to ER stress	- Vacuole formation inhibitors - Intracellular trafficking modulators - Organelle stabilizers
**2. Calcium Signaling Disruption [71,72,73,74]**	Excessive Ca^2+^ Release from ER [71]	- IP3 receptors - Ryanodine receptors - SERCA pumps	- Increased IP3 production - IP3R activation and Ca^2+^ release - Ca^2+^-induced Ca^2+^ release via RyRs - Impaired SERCA function due to ATP depletion	- Sustained cytosolic Ca^2+^ elevation - ER Ca^2+^ store depletion - Mitochondrial Ca^2+^ overload	- Triggers ER stress - Exacerbates mitochondrial dysfunction - Activates Ca^2+^-dependent enzymes	- IP3R inhibitors (e.g., 2-APB) - RyR modulators - SERCA activators
	Enhanced Ca^2+^ Influx (SOCE) [72]	- STIM1 - Orai1 - TRPC channels	- ER Ca^2+^ depletion sensed by STIM1 - STIM1 oligomerization and translocation - Activation of Orai1 channels - Ca^2+^ influx from extracellular space	- Prolonged cytosolic Ca^2+^ elevation - Cellular energy depletion - Activation of Ca^2+^-dependent pathways	- Amplifies initial Ca^2+^ signaling disruption - Contributes to oxidative stress - Affects membrane potential	- SOCE inhibitors (e.g., GSK-7975A) - STIM1/Orai1 modulators - TRPC channel blockers
	Impaired Ca^2+^ Extrusion [73]	- PMCA pumps - Na+/Ca^2+^ exchangers - ATP	- ATP depletion impairs PMCA function - Reduced Na^+^/Ca^2+^ exchanger activity - Accumulation of cytosolic Ca^2+^	- Prolonged cytosolic Ca^2+^ elevation - Disruption of Ca^2+^ gradients - Cellular stress and dysfunction	- Exacerbates energy crisis - Contributes to oxidative stress - Affects membrane integrity	- PMCA activators - Na^+^/Ca^2+^ exchanger modulators - ATP supplementation strategies
	Mitochondrial Ca^2+^ Overload [74]	- Mitochondrial Ca^2+^ uniporter (MCU) - MPTP - Cyclophilin D	- Excessive Ca^2+^ uptake by MCU - Mitochondrial Ca^2+^ overload - MPTP opening - Mitochondrial swelling and dysfunction	- Impaired ATP production - Increased ROS generation - Cytochrome c release	- Central to mitochondrial dysfunction - Contributes to apoptosis initiation - Affects cellular energy status	- MCU inhibitors - MPTP inhibitors (e.g., cyclosporin A) - Mitochondrial Ca^2+^ buffering enhancers
**3. Mitochondrial Dysfunction [75,76,77,78]**	MPTP Opening [75]	- Cyclophilin D - ATP synthase - Voltage-dependent anion channel (VDAC)	- Ca^2+^ overload and oxidative stress trigger MPTP opening - Loss of mitochondrial membrane potential - Swelling of mitochondria - Release of proapoptotic factors	- Energy crisis - Increased ROS production - Initiation of cell death pathways	- Central to mitochondria-mediated apoptosis - Exacerbates oxidative stress - Affects Ca^2+^ homeostasis	- Cyclophilin D inhibitors - MPTP stabilizers - Mitochondrial membrane potential preservers
	ATP Depletion [76]	- Electron transport chain complexes - ATP synthase - ADP/ATP translocase	- Impaired electron transport - Reduced proton gradient - Decreased ATP synthesis - Impaired ATP export from mitochondria	- Cellular energy crisis - Impaired ion pump function - Disruption of cellular processes	- Affects all ATP-dependent processes - Exacerbates Ca^2+^ overload - Impairs cellular repair mechanisms	- ETC complex activators - ATP synthase modulators - Mitochondrial substrate supplementation
	ROS Overproduction [77]	- Complex I and III of ETC - Superoxide dismutase (SOD) - Glutathione peroxidase	- Electron leakage from ETC - Formation of superoxide radicals - Overwhelmed antioxidant defenses - Oxidative damage to mitochondrial components	- Oxidative damage to proteins, lipids, and DNA - Further impairment of mitochondrial function - Activation of stress response pathways	- Contributes to MPTP opening - Activates inflammatory pathways - Enhances ER stress	- Mitochondria-targeted antioxidants (e.g., MitoQ) - SOD mimetics - ETC electron leak inhibitors
	Cytochrome c Release [78]	- Bax/Bak - Bcl-2 - Cytochrome c - Apaf-1	- Proapoptotic Bax/Bak activation - Outer mitochondrial membrane permeabilization - Cytochrome c release into cytosol - Formation of apoptosome	- Initiation of intrinsic apoptosis pathway - Caspase activation - Propagation of cell death signals	- Central to apoptosis regulation - Influences inflammatory responses - Affects overall cell fate decisions	- Bcl-2 inhibitors/activators - Caspase inhibitors - Apoptosome formation inhibitors
**4. Endoplasmic Reticulum (ER) Stress [79,80,81,82]**	Unfolded Protein Response (UPR) Activation [79]	- BiP/GRP78 - PERK - IRE1α - ATF6	- Accumulation of misfolded proteins - BiP dissociation from ER stress sensors - Activation of PERK, IRE1α, and ATF6 pathways - Induction of UPR target genes	- Global protein synthesis attenuation - Upregulation of chaperones - Enhanced ER-associated degradation (ERAD)	- Modulates inflammatory responses - Influences autophagy regulation - Affects cell survival decisions	- Chemical chaperones (e.g., 4-PBA, TUDCA) - UPR modulators - Protein folding enhancers
	PERK Pathway [80]	- PERK - eIF2α - ATF4 - CHOP	- PERK dimerization and autophosphorylation - eIF2α phosphorylation - Selective translation of ATF4 - Induction of CHOP	- Global protein synthesis inhibition - Upregulation of stress response genes - Potential apoptosis induction via CHOP	- Affects cellular redox state - Modulates autophagy - Influences lipid metabolism	- PERK inhibitors - eIF2α dephosphorylation modulators - CHOP inhibitors
	IRE1α Pathway [81]	- IRE1α - XBP1 - TRAF2 - JNK	- IRE1α oligomerization and activation - XBP1 mRNA splicing - JNK activation via TRAF2 - Regulated IRE1-dependent decay (RIDD)	- Upregulation of ER chaperones and ERAD components - Activation of inflammatory pathways - Selective mRNA degradation	- Crosstalk with inflammatory signaling - Affects lipid metabolism - Modulates cell death pathways	- IRE1α RNase inhibitors - JNK inhibitors - XBP1 modulators
	ATF6 Pathway [82]	- ATF6 - S1P and S2P proteases - ERAD components	- ATF6 translocation to Golgi - Cleavage by S1P and S2P - Nuclear translocation of cleaved ATF6 - Transcription of UPR target genes	- Increased ER folding capacity - Enhanced ERAD - Expansion of ER membrane	- Affects lipid biosynthesis - Modulates inflammatory responses - Influences cellular adaptation to stress	- ATF6 activators/inhibitors - S1P/S2P modulators - ERAD enhancers
**5. Autophagy Impairment [83,84,85,86]**	Initiation Defects [83]	- ULK1 complex - mTORC1 - AMPK	- Dysregulation of mTORC1/AMPK signaling - Impaired ULK1 complex activation - Reduced autophagosome formation initiation	- Accumulation of cellular debris - Impaired stress response - Reduced cellular quality control	- Affects cellular energy sensing - Influences ER stress responses - Modulates inflammatory pathways	- mTOR inhibitors (e.g., rapamycin) - AMPK activators - ULK1 activators
	Autophagosome Formation Defects [84]	- Beclin-1/VPS34 complex - ATG proteins (e.g., ATG5, ATG7) - LC3	- Impaired nucleation of phagophore - Defective elongation of autophagosomal membrane - Reduced LC3 lipidation	- Inefficient sequestration of cargo - Accumulation of protein aggregates and damaged organelles - Cellular stress amplification	- Affects mitochondrial quality control - Influences ER stress resolution - Modulates inflammatory responses	- Beclin-1/VPS34 activators - ATG protein modulators - LC3 lipidation enhancers
	Lysosomal Dysfunction [85]	- v-ATPase - Lysosomal hydrolases - LAMP proteins	- Impaired lysosomal acidification - Reduced hydrolase activity - Defective lysosomal membrane integrity	- Accumulation of autophagosomes - Inefficient degradation of cellular components - Potential release of lysosomal contents	- Exacerbates ER stress - Contributes to inflammatory activation - Affects cellular metabolism	- v-ATPase activators - Lysosomal membrane stabilizers - Hydrolase replacement therapies
	Autophagosome–Lysosome Fusion Defects [86]	- SNARE proteins - Rab7 - HOPS complex	- Impaired tethering of autophagosomes to lysosomes - Defective SNARE complex formation - Reduced fusion efficiency	- Accumulation of autophagosomes - Inefficient completion of autophagic flux - Cellular stress due to incomplete degradation	- Affects vesicular trafficking - Influences protein and organelle turnover - Modulates cellular homeostasis	- Rab7 activators - SNARE complex modulators - HOPS complex enhancers
**6. Inflammatory Response [87,88,89,90]**	DAMPs Release [87]	- HMGB1 - ATP - DNA - Heat shock proteins	- Cellular damage/necrosis - Release of intracellular components - Recognition by pattern recognition receptors (PRRs)	- Activation of innate immune responses - Initiation of sterile inflammation - Amplification of tissue damage	- Triggers TLR signaling - Activates NLRP3 inflammasome - Promotes neutrophil extracellular traps (NETs)	- DAMP neutralizing antibodies - TLR antagonists - HMGB1 inhibitors
	TLR Activation [88]	- TLR2, TLR4, TLR9 - MyD88 - TRIF	- DAMP recognition by TLRs - Recruitment of adaptor proteins - Activation of downstream signaling cascades	- NF-κB and AP-1 activation - Proinflammatory cytokine production - Leukocyte recruitment	- Amplifies inflammatory signaling - Influences cell death decisions - Modulates adaptive immune responses	- TLR antagonists - MyD88 inhibitors - NF-κB pathway modulators
	Inflammasome Activation [89]	- NLRP3 - ASC - Caspase-1 - IL-1β, IL-18	- Priming step: NF-κB-mediated upregulation of NLRP3 and pro-IL-1β - Activation step: NLRP3 oligomerization and inflammasome assembly - Caspase-1 activation and cytokine processing	- Release of mature IL-1β and IL-18 - Pyroptosis induction - Amplification of inflammatory responses	- Crosstalk with TLR signaling - Influences neutrophil recruitment - Affects adaptive immunity	- NLRP3 inhibitors - Caspase-1 inhibitors - IL-1 receptor antagonists
	Neutrophil Infiltration [90]	- Chemokines (e.g., IL-8) - Adhesion molecules - Neutrophil granule proteins	- Chemokine-guided migration - Adhesion to endothelium - Transmigration into tissue - Release of inflammatory mediators and NETs	- Tissue damage via proteases and ROS - Amplification of inflammatory signals - Potential microvascular occlusion	- Contributes to oxidative stress - Enhances vascular permeability - Modulates adaptive immune responses	- Chemokine receptor antagonists - Adhesion molecule inhibitors - NET inhibitors
**7. Cell Death [91,92,93,94]**	Apoptosis [91]	- Caspases (8, 9, 3, 7) - Bcl-2 family proteins - Cytochrome c - Apaf-1	- Extrinsic pathway: death receptor activation - Intrinsic pathway: mitochondrial outer membrane permeabilization - Caspase cascade activation - Controlled cellular dismantling	- Controlled cell death without inflammation - Maintenance of membrane integrity - Efficient clearance by phagocytes	- Influenced by ER stress and mitochondrial dysfunction - Modulates inflammatory responses - Affects tissue repair processes	- Caspase inhibitors - Bcl-2 family modulators - Death receptor antagonists
	Necrosis [92]	- RIPK1, RIPK3 - MLKL - Cyclophilin D	- Cellular stress or damage beyond repair capacity - ATP depletion and ion pump failure - Cellular swelling and membrane rupture - Release of cellular contents	- Uncontrolled cell death with inflammation - Release of DAMPs - Tissue architecture disruption	- Exacerbates inflammatory responses - Triggers adaptive immune activation - Affects surrounding healthy tissue	- Necrosis inhibitors - Cellular energy preservers - Membrane stabilizers
	Necroptosis [93]	- RIPK1, RIPK3 - MLKL - FADD, caspase-8	- Death receptor activation in absence of caspase-8 activity - RIPK1-RIPK3 necrosome formation - MLKL phosphorylation and oligomerization - Membrane permeabilization	- Programmed necrotic cell death - Inflammatory response induction - Potential pathogen clearance	- Crosstalk with apoptosis pathways - Modulates inflammatory signaling - Influences tissue damage extent	- RIPK1 inhibitors (e.g., Necrostatin-1) - RIPK3 inhibitors - MLKL inhibitors
	Pyroptosis [94]	- Caspase-1, Caspase-11 - Gasdermin D - NLRP3 inflammasome	- Inflammasome activation - Caspase-1/11 activation - Gasdermin D cleavage and pore formation - Cell lysis and IL-1β/IL-18 release	- Inflammatory form of programmed cell death - Cytokine release and inflammation amplification - Potential pathogen clearance	- Closely linked to inflammasome activation - Amplifies inflammatory responses - Affects tissue integrity	- Caspase-1 inhibitors - Gasdermin D inhibitors - IL-1 receptor antagonists
**8. Oxidative and Nitrosative Stress [95,96,97,98]**	Mitochondrial ROS Production [95]	- Complexes I and III of ETC - Superoxide dismutase (SOD) - Glutathione peroxidase	- Electron leakage from ETC - Superoxide radical formation - Conversion to H_2_O_2_ by SOD - Detoxification by glutathione system	- Oxidative damage to mitochondrial components - mtDNA mutations - Impaired ATP production	- Exacerbates mitochondrial dysfunction - Triggers MPTP opening - Activates stress response pathways	- Mitochondria-targeted antioxidants - ETC modulators - SOD mimetics
	NADPH Oxidase Activation [96]	- NOX enzymes - p47phox, p67phox - Rac proteins	- Assembly of NOX complex at membrane - Electron transfer to molecular oxygen - Superoxide production - Conversion to other ROS species	- Extracellular and phagosomal ROS production - Oxidative damage to cellular components - Activation of redox-sensitive pathways	- Contributes to neutrophil-mediated damage - Modulates inflammatory signaling - Affects vascular function	- NOX inhibitors - Assembly inhibitors - ROS scavengers
	Xanthine Oxidase Activation [97]	- Xanthine dehydrogenase - Xanthine oxidase - Hypoxanthine/xanthine	- Conversion of xanthine dehydrogenase to oxidase - Hypoxanthine/xanthine oxidation - Superoxide and H_2_O_2_ production - Uric acid formation	- Increased ROS during ischemia reperfusion - Oxidative damage to cellular components - Potential NLRP3 inflammasome activation	- Exacerbates ischemia-reperfusion injury - Contributes to vascular dysfunction - Modulates inflammatory responses	- Xanthine oxidase inhibitors (e.g., allopurinol) - Antioxidants - Uric acid lowering agents
	Nitrosative Stress [98]	- iNOS - Peroxynitrite - Nitrotyrosine	- iNOS upregulation and activation - Excessive NO production - Reaction with superoxide to form peroxynitrite - Protein tyrosine nitration	- Nitrosative modification of proteins - DNA and lipid damage - Mitochondrial dysfunction	- Interacts with oxidative stress pathways - Modulates cellular signaling - Affects enzyme function and protein stability	- iNOS inhibitors - Peroxynitrite scavengers - Protein denitration strategies
**9. Microcirculatory Dysfunction [99,100,101,102]**	Vasoconstriction [99]	- Endothelin-1 - Thromboxane A2 - Angiotensin II	- Release of vasoconstrictors - Smooth muscle contraction - Reduced vessel diameter - Decreased blood flow	- Tissue ischemia - Impaired nutrient and oxygen delivery - Exacerbation of cellular stress	- Contributes to oxidative stress - Affects inflammatory cell recruitment - Modulates tissue edema	- Endothelin receptor antagonists - Thromboxane inhibitors - Vasodilators
	Increased Vascular Permeability [100]	- VEGF - Bradykinin - Histamine - Leukotrienes	- Release of permeability factors - Endothelial cell contraction - Tight junction disruption - Increased paracellular transport	- Tissue edema - Fluid sequestration - Potential compartment syndrome	- Exacerbates inflammatory responses - Affects drug delivery to tissue - Modulates immune cell extravasation	- VEGF inhibitors - Bradykinin receptor antagonists - Antihistamines
	Leukocyte–Endothelial Interactions [101]	- Selectins (P, E, L) - Integrins - ICAM-1, VCAM-1	- Leukocyte rolling (selectins) - Firm adhesion (integrins) - Transmigration - Release of inflammatory mediators	- Increased inflammatory cell infiltration - Endothelial activation and dysfunction - Microvascular occlusion	- Amplifies local inflammation - Contributes to tissue damage - Affects microvascular blood flow	- Selectin inhibitors - Integrin antagonists - Adhesion molecule blockers
	Microthrombi Formation [102]	- Tissue factor - Platelets - Fibrin - von Willebrand factor	- Tissue factor exposure - Platelet activation and aggregation - Fibrin deposition - Thrombus formation	- Microvascular occlusion - Tissue ischemia - Potential organ dysfunction	- Interacts with coagulation cascades - Affects inflammatory responses - Modulates tissue repair processes	- Anticoagulants - Antiplatelet agents - Fibrinolytic therapies
**10. Genetics [103,104,105,106]**	*PRSS1* Mutations [103]	- Cationic trypsinogen - Trypsin	- Gain-of-function mutations in PRSS1 - Enhanced trypsinogen autoactivation - Resistance to protective mechanisms - Increased trypsin activity	- Increased susceptibility to pancreatitis - Enhanced acinar cell injury - Chronic inflammation and fibrosis	- Amplifies premature enzyme activation - Affects cellular stress responses - Modulates inflammatory pathways	- Personalized trypsin inhibitors - Gene therapy approaches - Pancreatic enzyme replacement
	*SPINK1* Mutations [104]	- Pancreatic secretory trypsin inhibitor	- Loss-of-function mutations in *SPINK1* - Reduced trypsin inhibition capacity - Imbalance in protease–antiprotease equilibrium - Enhanced trypsin activity	- Increased risk of pancreatitis - Exacerbation of acinar cell damage - Potential progression to chronic pancreatitis	- Interacts with trypsin activation pathways - Affects ER stress responses - Modulates inflammatory intensity	- SPINK1 supplementation strategies - Alternative protease inhibitors - Targeted anti-inflammatory approaches
	*CFTR* Mutations [105]	- Cystic fibrosis transmembrane conductance regulator	- Impaired *CFTR* function - Altered ductal secretion - Changes in pancreatic juice composition - Potential protein precipitation in ducts	- Increased risk of pancreatitis - Ductal obstruction - Potential progression to pancreatic insufficiency	- Affects fluid and bicarbonate secretion - Modulates acinar–ductal interactions - Influences inflammatory responses	- *CFTR* modulators/potentiators - Mucolytic therapies - Ductal function enhancers
	*CTRC* Mutations [106]	- Chymotrypsin C	- Loss-of-function mutations in *CTRC* - Impaired trypsin degradation - Prolonged trypsin activity - Enhanced risk of trypsin-mediated damage	- Increased susceptibility to pancreatitis - Exacerbation of acinar cell injury - Potential chronic inflammation	- Interacts with trypsin activation/inactivation pathways - Affects protease–antiprotease balance - Modulates cellular stress responses	- *CTRC* replacement strategies - Alternative trypsin degradation enhancers - Targeted protease inhibitors

ATP—adenosine triphosphate; ADP—adenosine diphosphate; ASC—apoptosis-associated speck-like protein containing a CARD; ATF4—activating transcription factor 4; ATF6—activating transcription factor 6; Bcl-2—B-cell lymphoma 2; BiP—binding immunoglobulin protein; CFTR—cystic fibrosis transmembrane conductance regulator; CHOP—C/EBP homologous protein; CINC—cytokine-induced neutrophil chemoattractant; CTRC—chymotrypsin C; DAMPs—damage-associated molecular patterns; DNA—deoxyribonucleic acid; ER—endoplasmic reticulum; ETC—electron transport chain; ERAD—ER-associated degradation; FADH—flavin adenine dinucleotide; GRP78—glucose-regulated protein 78; H_2_O_2_—hydrogen peroxide; HMGB1—high mobility group box 1; HOPS—homotypic fusion and protein sorting; ICAM-1—intercellular adhesion molecule 1; IL—interleukin; iNOS—inducible nitric oxide synthase; IP3R—inositol 1,4,5-trisphosphate receptor; IRE1α—inositol-requiring enzyme 1α; JNK—c-Jun N-terminal kinase; LAMP—lysosomal-associated membrane protein; LC3—microtubule-associated protein 1A/1B-light chain 3; MCU—mitochondrial calcium uniporter; MLKL—mixed lineage kinase domain-like protein; MPTP—mitochondrial permeability transition pore; mTORC1—mammalian target of papamycin complex 1; MyD88—myeloid differentiation primary response 88; NADPH—nicotinamide adenine dinucleotide phosphate; NETs—neutrophil extracellular traps; NF-κB—nuclear factor kappa-light-chain-enhancer of activated B cells; NLRP3—NOD-, LRR-, and pyrin-domain-containing protein 3; NO—nitric oxide; NOX—NADPH oxidase; PERK—protein kinase R (PKR)-like endoplasmic reticulum kinase; PMCA—plasma membrane Ca^2+^ ATPase; PRRs—pattern recognition receptors; PRSS1—protease serine 1 (cationic trypsinogen); PSTI—pancreatic secretory trypsin inhibitor; RIPK—receptor-interacting serine/threonine-protein kinase; ROS—reactive oxygen species; RyR—ryanodine receptor; S1P—site-1 protease; S2P—site-2 protease; SERCA—sarco-/endoplasmic reticulum Ca2+-ATPase; SNARE—soluble N-ethylmaleimide-sensitive factor attachment protein receptor; SOD—superoxide dismutase; SPINK1—serine protease inhibitor Kazal type 1; STIM1—stromal interaction molecule 1; TLR—toll-like receptor; TNF-α—tumor necrosis factor alpha; TRAF2—TNF receptor-associated factor 2; TRIF—TIR-domain-containing adapter-inducing interferon-β; ULK1—Unc-51-like autophagy-activating kinase 1; UPR—unfolded protein response; VCAM-1—vascular cell adhesion molecule 1; VEGF—vascular endothelial growth factor; VPS34—vacuolar protein sorting 34; XBP1—X-box binding protein 1.

## Data Availability

Data available on request.
